# Leukemic conversion involving *RAS* mutations of type 1 *CALR*-mutated primary myelofibrosis in a patient treated for HCV cirrhosis: a case report

**DOI:** 10.3389/fonc.2023.1266996

**Published:** 2023-09-29

**Authors:** Petruta Gurban, Cristina Mambet, Anca Botezatu, Laura G. Necula, Ana I. Neagu, Lilia Matei, Ioana M. Pitica, Saviana Nedeianu, Mihaela Chivu-Economescu, Coralia Bleotu, Marius Ataman, Gabriela Mocanu, Carmen Saguna, Anca G. Pavel, Danae Stambouli, Elise Sepulchre, Gabriela Anton, Carmen C. Diaconu, Stefan N. Constantinescu

**Affiliations:** ^1^ Cellular and Molecular Pathology Department, Stefan S. Nicolau Institute of Virology, Romanian Academy, Bucharest, Romania; ^2^ Cytogenomic Medical Laboratory Ltd., Bucharest, Romania; ^3^ Department of Radiology, Oncology, and Hematology, Faculty of Medicine, Carol Davila University of Medicine and Pharmacy, Bucharest, Romania; ^4^ Hematology Department, Emergency University Clinical Hospital, Bucharest, Romania; ^5^ Molecular Virology Department, Stefan S. Nicolau Institute of Virology, Romanian Academy, Bucharest, Romania; ^6^ Department of Hematology, Coltea Clinical Hospital, Bucharest, Romania; ^7^ De Duve Institute, Université Catholique de Louvain, Brussels, Belgium; ^8^ SIGN (Cell Signalling and Molecular Hematology), Ludwig Institute for Cancer Research Brussels, Brussels, Belgium; ^9^ Walloon Excellence in Life Sciences and Biotechnology (WELBIO) Department, WEL Research Institute, Wavre, Belgium; ^10^ Nuffield Department of Medicine, Ludwig Institute for Cancer Research, Oxford University, Oxford, United Kingdom

**Keywords:** primary myelofibrosis (PMF), hepatitis C virus (HCV) cirrhosis, type 1 calreticulin (CALR), targeted next-generation sequencing (NGS), *NRAS*, acquired uniparental disomy (aUPD), case report

## Abstract

Somatic frameshift mutations in exon 9 of calreticulin (*CALR*) gene are recognized as disease drivers in primary myelofibrosis (PMF), one of the three classical Philadelphia-negative myeloproliferative neoplasms (MPNs). Type 1/type 1-like *CALR* mutations particularly confer a favorable prognostic and survival advantage in PMF patients. We report an unusual case of PMF incidentally diagnosed in a 68-year-old woman known with hepatitis C virus (HCV) cirrhosis who developed a progressive painful splenomegaly, without anomalies in blood cell counts. While harboring a type 1 *CALR* mutation, the patient underwent a leukemic transformation in less than 1 year from diagnosis, with a lethal outcome. Analysis of paired DNA samples from chronic and leukemic phases by a targeted next-generation sequencing (NGS) panel and single-nucleotide polymorphism (SNP) microarray revealed that the leukemic clone developed from the *CALR*-mutated clone through the acquisition of genetic events in the RAS signaling pathway: an increased variant allele frequency of the germline *NRAS* Y64D mutation present in the chronic phase (via an acquired uniparental disomy of chromosome 1) and gaining *NRAS* G12D in the blast phase. SNP microarray analysis showed five clinically significant copy number losses at regions 7q22.1, 8q11.1-q11.21, 10p12.1-p11.22, 11p14.1-p11.2, and Xp11.4, revealing a complex karyotype already in the chronic phase. We discuss how additional mutations, detected by NGS, as well as HCV infection and antiviral therapy, might have negatively impacted this type 1 *CALR*-mutated PMF. We suggest that larger studies are required to determine if more careful monitoring would be needed in MPN patients also carrying HCV and receiving anti-HCV treatment.

## 1 Introduction

Primary myelofibrosis (PMF) is the most severe disease subtype of the classical *BCR-ABL1-*negative myeloproliferative neoplasms (MPNs), which include also polycythemia vera (PV) and essential thrombocythemia (ET) (1). It is a hematopoietic stem cell (HSC) clonal disorder defined by specific histologic anomalies in bone marrow (BM) biopsy, extramedullary hematopoiesis, abnormal cytokine production, clinical heterogeneity, and an increased risk of transformation into secondary acute myeloid leukemia (sAML) (2). According to the criteria of the International Consensus Classification of Myeloid Neoplasms and Acute Leukemias, PMF is further subdivided into “prefibrotic” and “overt fibrotic” stages (3). Prefibrotic PMF is mainly characterized by thrombocytosis, mimicking ET, while fibrotic PMF usually displays anemia and leukocytosis, variable platelet (PLT) count, and a typical leukoerythroblastic blood smear (2).

At the molecular level, somatic activating driver mutations in Janus Kinase 2 (*JAK2*), thrombopoietin receptor (*MPL* and *TPOR*), and calreticulin (*CALR*) genes —the so-called MPN drivers— result in constitutive activation of the Janus Kinase and Signal Transducer and Activator of Transcription (JAK/STAT) pathway via MPL, which is responsible for clonal myeloproliferation in most cases (4). Additionally, other variants/mutations that affect epigenetic regulators, spliceosome components, signaling molecules, or transcription factors may cooperate with MPN drivers and influence the disease outcome (5). The information about molecular markers was incorporated into the recently developed PMF prognostic models, and *SRSF2*, *ASXL1*, *EZH2*, *IDH1*/*IDH2*, and *U2AF1*-Q157 mutations were identified as independent risk factors of poor outcome in PMF and thus considered high-molecular- risk (HMR) mutations (2, 6).

As MPN drivers, *CALR* mutations, represented by frameshift deletions, insertions, or complex indels within exon 9, are detected in 25% of PMF patients (2). CALR mutants that are characterized by a new C-terminal protein sequence (7) display oncogenic activity as they bind directly to MPL, leading to persistent receptor activation, JAK2-STAT5/STAT3/STAT1, MAP-kinase, and PI-3′kinase/Akt activation, and dysregulated megakaryopoiesis (8–12). The most prevalent *CALR* mutations are a 52-bp deletion (del 52, p.L367fs*46) and a 5-bp TTGTC insertion (ins5, p.K385fs*47) (7), known also as type 1 and type 2 *CALR* mutations, respectively. Based on the alpha-helix content in the secondary structure of the mutated protein and the remaining negatively charged region in the novel C-terminus, the other *CALR* variants are classified into type 1-like and type 2-like (13). This classification has prognostic relevance, as type 1/type 1-like mutations are associated with a favorable prognosis and survival advantage in PMF patients. Moreover, type 1/type 1-like *CALR* mutations partially ameliorate the deleterious effect of HMR mutations (14).

Leukemic conversion in PMF is encountered in 9%–13% of patients and is associated with a dismal prognosis. Risk factors for sAML development are represented by age above 70 years, moderate/severe anemia, thrombocytopenia, more than 3% circulating blasts, high risk/unfavorable karyotype, and the presence of *IDH1*/*IDH2*, *SRSF2*, or *ASXL1* mutation (15). Apart from genetic risk factors, chronic inflammation related to aberrant production of the pro-inflammatory cytokines and reactive oxygen species (ROS) in the BM microenvironment might be involved in disease progression to the blast phase (16).

There are no studies concerning the impact of chronic HCV infection and direct-acting antiviral drugs on MPN outcomes.

**Figure 1:**
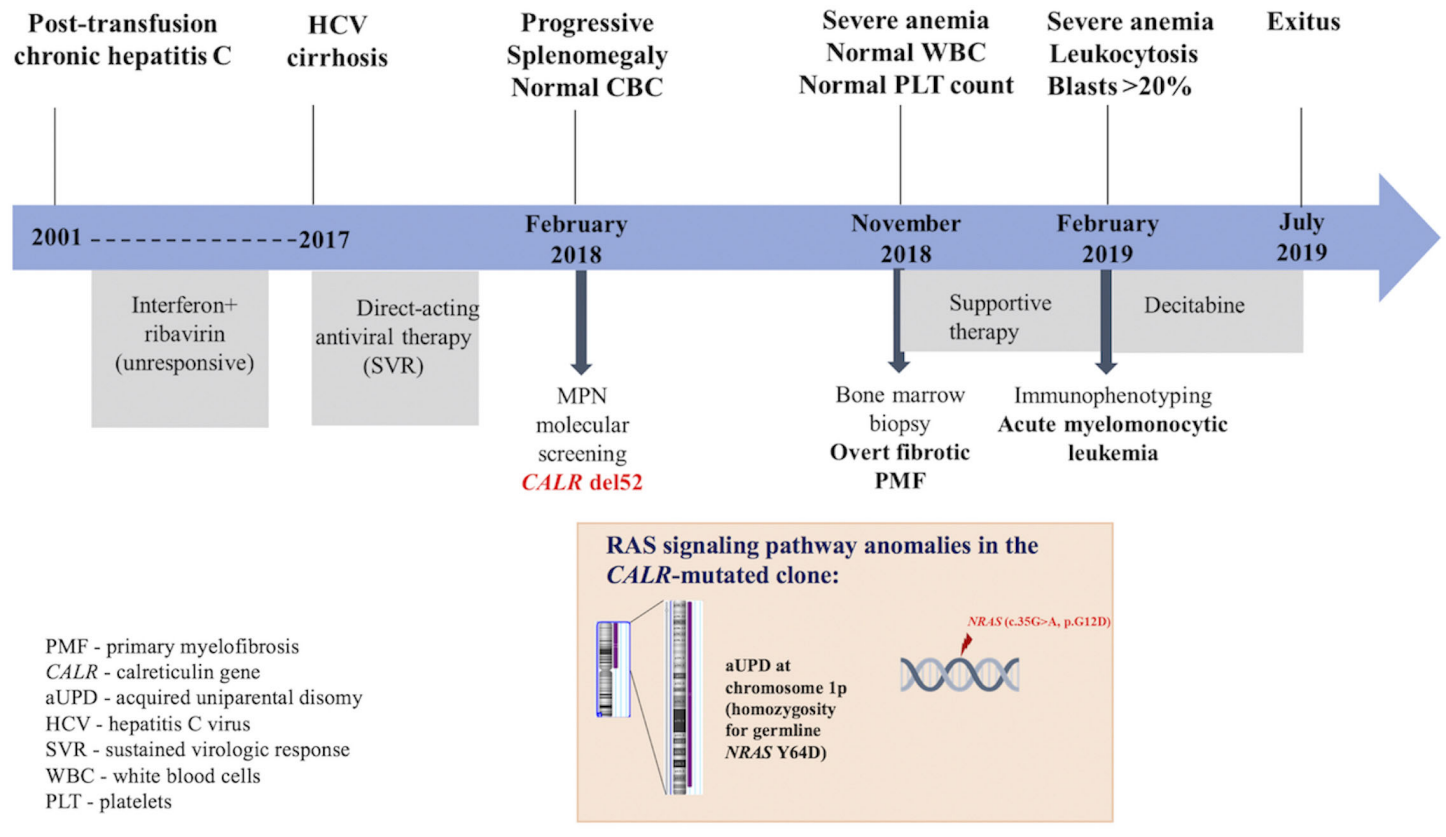
Diagram of patient’s medical history, genetic analysis, and therapeutic procedures.

In this report, we describe the case of an elderly female patient known with hepatitis C virus (HCV) infection since 2001 and treated with direct-acting antiviral drugs in 2017 for HCV cirrhosis, incidentally diagnosed with PMF and type 1 *CALR* mutation, who exhibited an accelerated progression to refractory sAML. We discuss how the additional pathogenic mutations in the *NRAS* gene identified by targeted next-generation sequencing (NGS), as well as HCV infection and antiviral therapy, might have contributed to the unfavorable disease outcome. A summary of the patient’s medical history and the genetic landscape is presented in **Figure 1**.

## 2 Case presentation

A 68-year-old woman with post-transfusion chronic HCV since 2001, unresponsive to conventional antiviral treatment, (interferon+ribavirine) and evolving to liver cirrhosis, obtained sustained virologic response after direct-acting antiviral therapy (ombitasvir + paritaprevir + ritonavir) in 2017. However, within 3 months, the patient developed painful splenomegaly. No abnormality in complete blood count (CBC) was observed, except for a mild normochromic normocytic anemia (Hb 11.0 g/dL). Previous CBC reports were normal as well, arguing against a secondary myelofibrosis condition. Although splenomegaly could have been associated with advanced liver fibrosis, a screening for MPN drivers was performed at referral to a hematologist in February 2018. The most common MPN driver mutation *JAK2* V617F was absent in granulocytic DNA when tested by allele-specific PCR. Furthermore, a type 1 *CALR* mutation (del 52) with a variant allele frequency (VAF) of 50.2% was detected by GeneScan fragment size analysis. At this stage, the patient did not give her consent for a BM biopsy. Eight months later, the patient complained of marked asthenia and fatigability. At presentation to the Hematology Clinic of “Coltea” Hospital, Bucharest, massive splenomegaly accompanied by severe anemia (Hb 6.5 g/dL), normal white blood cell (WBC), and PLT counts, were detected. Peripheral blood smear examination revealed the presence of erythroblasts (10 per 100 WBCs), and BM biopsy was suggestive of overt fibrotic PMF (MF-2). Only supportive treatment was given. After 3 months, progression to sAML was observed: WBC of 24.1 × 10^9^/L with an increased percentage (40%) of polymorphic blasts, 1% promyelocytes, 2% myelocytes, 10% metamyelocytes, 30% neutrophils, 1% eosinophils, 4% basophils, 4% lymphocytes, and 8% monocytes, accompanied by severe anemia (Hb 5.8 g/dL) and normal PLT count. Immunophenotyping by flow cytometry of BM aspirate revealed a blast cell population including 15% myeloblasts (CD34+, HLADR+, CD13+, CD33+, and CD38+) and 25% promonocytes (CD64+, CD13+, CD33+, HLADR+, and aberrant expression of CD56), a profile compatible with acute myelomonocytic leukemia. Conventional cytogenetic examination could not be performed. The patient received three cycles of decitabine (20 mg/m^2^ per day for five consecutive days, repeated every 4 weeks). Unfortunately, a progressive increase in WBC was observed associated with tumor lysis syndrome that led to severe electrolyte and metabolic disturbances and ultimately to death as a result of multiple organ failure.

We selected this case for an in-depth study in order to gain insight into the molecular mechanisms responsible for the disease progression. The research was conducted in compliance with the Declaration of Helsinki and was approved by the local ethics committee (No. 136/06.02.2017 rev. no.131/18.01.2019). A separate written informed consent was obtained from the patient at the moment of blood and bone marrow collection for the genetic analyses performed at Stefan S. Nicolau Institute of Virology. Details concerning the methods are found in the Supplementary Material. For all subsequent analyses, specific information was de-identified. Details concerning the methods are found in the **Supplementary Material**.

Peripheral blood mononuclear cells (PBMC) and bone marrow mononuclear cells (BMMC) were obtained by density gradient centrifugation using Ficoll-Paque under standard procedures. Granulocytes were separated after the collection of PBMC and red cell lysis of the pellet with a hypotonic solution. CD34+ and CD3+ cells were isolated immunomagnetically from BMMC. DNA was extracted from all cell fractions.

Detection of *CALR* exon 9 mutations in granulocytes and CD34+ and CD3+ cells was performed by GeneScan DNA fragment size analysis as previously described (17). Furthermore, paired DNA samples from chronic and leukemic phases were analyzed with TruSight Myeloid Sequencing Panel (Illumina, San Diego, CA, USA), an amplicon-based NGS panel that targets the full exonic regions of 15 genes and exonic hot spots of 39 genes with known relevance for myeloid malignancies. Thus, in addition to *CALR* del52, *NRAS* (c.190T>G, p.Y64D) and *PTPN11* (c.1504T>A, p.S502T) missense mutations were detected in granulocytic DNA obtained at initial presentation, with a VAF of 44.8% and 21.4%, respectively. At the moment of leukemic conversion, the DNA samples used for NGS testing were extracted from two cell fractions isolated from BMMC— CD34+ cells to include a part of the blast cell population and CD3+ T cells to serve as non-tumoral tissue control— as previously suggested for molecular analysis in MPN patients (18). *CALR* del52 was detected by GeneScan fragment size analysis in CD34+ cells with a similar VAF (51%) as those from the chronic phase, while in CD3+ cells, the MPN driver mutation was present at a lower VAF (24.3%). Targeted NGS on CD34+ cells revealed a higher VAF for the previously detected *NRAS* Y64D mutation (90.9%), indicating an evolution to homozygosity, along with a newly acquired *NRAS* mutation (c.35G>A, p.G12D), known as pathogenic in many types of cancers, including myeloid neoplasms (19), displaying a VAF of 43%. The *PTPN11* mutation was no longer detected. A similar analysis of CD3+ cells detected the presence of *NRAS* Y64D mutation with a VAF of 49%, thus confirming its germline origin, while *NRAS* G12D mutation was absent.

**Figure 2:**
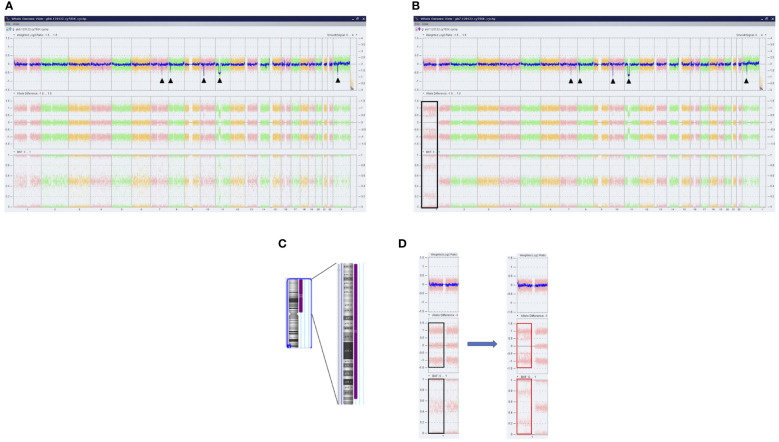
Results of SNP microarray analysis in PMF patient. Whole-genome analysis of SNVs and CN-LOH provided by ChAS software in DNA samples obtained at chronic phase **(A)** and leukemic conversion **(B)**. Each dot corresponds to a single SNP in the array. The DNA copy number losses (deletions/microdeletions) at the level of chromosomes 7q, 8q, 10p, 11p, and Xp are associated with a loss of signal intensity corresponding to a decrease in Weighted Log2 ratio (black arrows). In sample B, the CN-LOH on chromosome 1 that was acquired at leukemic transformation is shown (black box). **(C)** Ideogram of chromosome 1 and the region of CN-LOH indicated to the right in purple; also, the affected cytobands are shown. **(D)** Magnified image of SNP array results of chromosome 1 before and after the occurrence of UPD. No changes in Weighted Log2 ratio are observed. Allele difference and B allele frequency (BAF) plots indicate the presence of two tracks (red boxes) instead of the normal three tracks (black boxes), consistent with the loss of heterozygosity. SNP, single-nucleotide polymorphism; SNVs, single-nucleotide variants; CN-LOH, copy neutral loss of heterozygosity; UPD, uniparental disomy.

**Figure 3:**
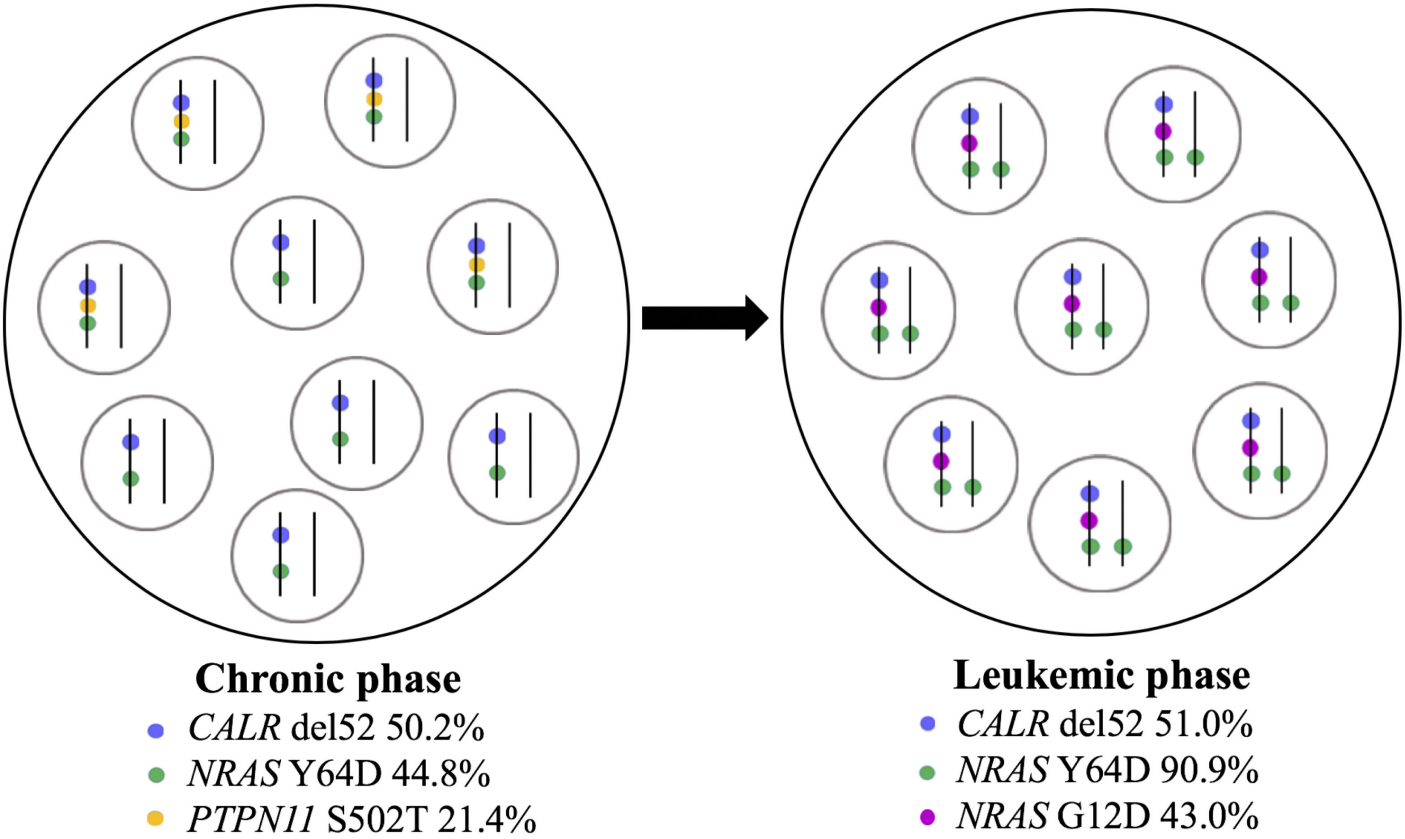
Schematic representation of clonal evolution in PMF patient. *CALR* del52, *NRAS* Y64D, and *PTPN11* S502 mutations were detected at diagnosis in peripheral blood granulocytes in a heterozygous state according to VAF. At leukemic stage, *CALR* del52 was detected with a similar VAF in CD34+ cells, *NRAS* Y64D mutation suffered a transition to a homozygous state, *PTPN11* mutation was no longer found, and a new *NRAS* mutation (G12D) was acquired. The leukemic clone arose from the *CALR*-mutated ancestral clone. Each circle contains a population of cells; in each cell, mutant alleles are represented by colored points on vertical parallel lines to suggest heterozygous versus homozygous state. PMF, primary myelofibrosis; VAF, variant allele frequency.

Furthermore, a high-resolution genome-wide single-nucleotide polymorphism (SNP) microarray was employed to test for the presence of chromosomal aberrations at the level of the whole genome such as copy number variations (CNVs) and copy-neutral loss of heterozygosity (CN-LOH), the latter known also as uniparental disomy (UPD). Paired DNA samples were analyzed for targeted NGS; however, the DNA sample from the blast phase was obtained from PBMC due to the low amount of CD34+ cell DNA. Also, the amount of CD3+ cell DNA was insufficient for the SNP array testing. In both samples, five clinically significant copy number losses according to Online Mendelian Inheritance in Man (OMIM) were detected at regions 7q22.1, 8q11.1-q11.21, 10p12.1-p11.22, 11p14.1-p11.2, and Xp11.4, revealing a complex karyotype (**Figures 2A, B**). Additionally, an acquired UPD (aUPD) at 1p36.3-1p12 (118 Mb), covering almost the entire length of the short arm of chromosome 1, was identified only in the sample obtained at the moment of leukemic conversion (**Figure 2C**). The CN-LOH observed at chromosome 1p is partial, being suggestive of a mosaic aUPD (**Figure 2D**). The UPD region comprises 1388 OMIM genes, including also *NRAS* gene at 1p13.2. Thus, the increase in *NRAS* Y64D mutational load observed in the leukemic phase sample is related to the occurrence of an aUPD at 1p, as shown previously for other mutations (20). The mosaic pattern of aUPD is most likely due to the fact that the second DNA sample was obtained from PBMC, which includes, apart from myeloid blasts, lymphocytes T, lymphocytes B, natural killer cells, monocytes, and dendritic cells (21). Based on the similar VAF (approximately 50%) of *CALR* mutation detected in the paired DNA samples, indicating a heterozygous status, it may be assumed that the leukemic clone evolved from the *CALR*-mutated clone through acquisition of a segmental UPD at chromosome 1p that led to homozygosity of initially detected *NRAS* Y64D mutation and of an additional *NRAS* G12D mutation (**Figure 3**).

## 3 Discussion

We describe a case of fibrotic PMF that at the first presentation lacked the characteristic CBC anomalies and the leukoerythroblastic blood smear. The suspicion of MPN was raised due to the progressive painful splenomegaly that could not be explained solely by the compensated HCV cirrhosis. The workup for MPN drivers revealed a type 1 *CALR* mutation. The PMF diagnosis was established when severe anemia was installed, and the patient gave her consent to a BM biopsy.

Cases of MPNs with normal CBC were mostly reported in association with splanchnic vein thrombosis, with *JAK2* V617F mutation being highly prevalent in this condition, although at low allele burden. Portal hypertension resulting in splenomegaly with hypersplenism and hemodilution likely explains the normal blood results (22). A single case of MPN without CBC anomalies was described in a completely asymptomatic young patient who presented with splenomegaly during a regular check-up (23). Molecular analysis detected the *JAK2* V617F mutation, and BM biopsy revealed megakaryocytic hyperplasia with dysplastic changes and a focal increase of reticulin fibers (23).

As was shown previously (24), *CALR* mutations represent the first genetic event in MPN cases, conferring an early clonal dominance in the HSC compartment. Compared to *CALR* type 2, type 1 *CALR* mutations penetrate faster in the T lymphocyte lineage. In our case, this might explain the presence of *CALR* del52 in CD3+ cells with a VAF of 24.3%, compared to a complete penetrance in granulocytes (VAF of approximately 50%).

The disease conversion into sAML that ended up with patient exitus occurred after the incidental discovery of PMF in the context of a normal CBC and the presence of type 1 *CALR* as an MPN driver mutation. However, it is important to mention that despite being associated with increased overall survival in comparison with the other MPN driver mutations, the presence of type 1 *CALR* does not influence the risk of leukemic conversion (14). To look for additional mutations, paired DNA samples from chronic and blastic phases were analyzed by targeted NGS, and anomalies in the RAS signaling pathway represented by two missense *NRAS* mutations were detected. It is known that missense *RAS* mutations that are reported in various human malignancies are gain-of-function mutations that result in a constitutive activation of RAS proteins (25). RAS activation will generate an increase in signaling through RAF1 (rapidly accelerated fibrosarcoma 1)–MEK (mitogen-activated protein kinase kinase)–ERK (extracellular signal-regulated kinase) and phosphoinositide-3 kinase (PI3K)/AKT (protein kinase B) pathways (26). Concerning the prognostic impact of *RAS* mutations, in a recent study that included 723 patients with PMF and post-PV or post-ET myelofibrosis (MF), oncogenic *NRAS*/*KRAS* mutations conferred a high risk of developing sAML, leading to reduced survival. These patients may benefit from dual therapy with JAK2 and MEK inhibitors (27). Oncogenic *NRAS* mutations are frequently reported in myeloid neoplasms associated with monocyte lineage involvement. In a murine BM transplantation model carrying *NRAS* G12D, a myeloproliferative disease similar to chronic myelomonocytic leukemia (CMML) was induced after a prolonged latency period. An aberrant granulocyte-macrophage colony-stimulating factor signaling was detected in granulocytic/monocytic precursors (28). Also, in a bone marrow transduction and transplantation mouse model, it was shown that the oncogenic *NRAS* mutation was able to induce both CMML and AML-like diseases (mainly acute monocytic anemia), with AML being found to have a higher level of *NRAS* expression (29).

Compared to *NRAS* G12D, the biological significance of *NRAS* Y64D mutation that proved to be a germline variant is not clear. According to FATHMM-MKL (30) and SIFT (31) algorithms, a deleterious effect of this mutation is predicted. On the contrary, in a functional study that employed a pooled *in vivo* screen, Y64D mutant did not promote tumor formation, and it induced a gene expression profile in culture close to the signature of the wild-type *NRAS* allele (32). On the contrary, in an *in vitro* cellular assay, *NRAS* mutant on the amino acid residue Y64 caused an interleukin-3- independent growth of Ba/F3 cells (33). Unfortunately, functional studies to assess the pathogenicity of *NRAS* Y64D mutation in our case could not be performed. It is possible that by acquiring a homozygous state via UPD1p, *NRAS* Y64D mutation might have contributed to leukemogenesis in cooperation with *NRAS* G12D mutation. The occurrence of two different *NRAS* mutations in the same patient was described only in three cases of *de novo* AML (34).

The prognostic impact of the complex karyotype that was present at MPN diagnosis in our case cannot be overlooked, too. According to the revised cytogenetic risk stratification in PMF, the complex karyotype —at least three distinct numeric or structural chromosome abnormalities —is assigned to an unfavorable category associated with a median survival of 2.9 years (35).

Apart from the negative impact of *NRAS* mutations and complex karyotype, particularly in this case, we could not disregard mentioning the occurrence of HCV infection and antiviral therapy, especially in the context of the complex karyotype detected at the patient’s first presentation at the hematologist when CBC results were still normal. Nevertheless, our study could not investigate the involvement of HCV infection or antiviral drugs in the leukemic transformation of this PMF case, as no DNA samples from the period of HCV infection monitoring were available to test for the presence of type 1 *CALR* mutation. Of interest, it was demonstrated that HCV infection leads to a defective DNA damage repair by increased production of ROS and nitrogen species, as well as by disrupting the DNA binding of essential repair enzymes. These defects are able to generate chromosomal alterations in both hepatocytes and PBMC (36). Moreover, HCV can infect CD34+ hematopoietic progenitors in chronic carriers (37), and in a large epidemiologic study, HCV infection was associated with an elevated risk of developing AML (38). Also, treatment with directly acting anti-HCV drugs was found to induce genomic instability (39). Further studies are required to establish a link between HCV infection or antivirals and the progression of MPNs.

In summary, the PMF case presented is characterized by complex clinical and genetic features. Despite the low clinical suspicion of a myeloproliferative disorder, due to normal blood counts and associated HCV cirrhosis, screening for MPN drivers was employed and identified a type 1 *CALR* mutation. At the moment of PMF diagnosis, a poor clinical outcome was not expected, as type 1 *CALR* mutation is considered a favorable prognostic factor for PMF, although the precise rates of transformation to AML need to be established on large cohorts. The retrospective comprehensive molecular analysis revealed significant CNV anomalies in addition to the MPN driver that, perhaps in the context of HCV infection and antiviral treatment, may have contributed to the accelerated disease evolution to refractory sAML. We underscore the importance of advanced molecular assays as an essential tool for prognostic assessment in PMF and also for guiding future targeted therapy. Finally, we suggest that patients with PMF/MPNs and simultaneous HCV infection and antiviral treatment be closely monitored for progression. Our case underscores the need for larger studies on this type of patients.

## Data availability statement

The datasets presented in this article are not readily available because of ethical/privacy restrictions. Requests to access the datasets should be directed to the corresponding author.

## Ethics statement

The studies involving humans were approved by Stefan S. Nicolau Institute of Virology Institutional Review Board. The studies were conducted in accordance with the local legislation and institutional requirements. The participants provided their written informed consent to participate in this study. Written informed consent was obtained from the individual(s) for the publication of any potentially identifiable images or data included in this article.

## Author contributions

PG: Conceptualization, Methodology, Writing – original draft, Writing – review & editing, Data curation, Formal Analysis, Investigation, Visualization. CM: Conceptualization, Data curation, Formal Analysis, Investigation, Methodology, Validation, Visualization, Writing – original draft, Writing – review & editing. AB: Data curation, Formal Analysis, Investigation, Methodology, Visualization, Writing – original draft. LN: Data curation, Formal Analysis, Investigation, Methodology, Validation, Visualization, Writing – original draft. AN: Data curation, Formal Analysis, Investigation, Methodology, Validation, Visualization, Writing – original draft. LM: Data curation, Formal Analysis, Investigation, Methodology, Validation, Visualization, Writing – review & editing. IP: Data curation, Formal Analysis, Investigation, Methodology, Validation, Visualization, Writing – original draft. SN: Data curation, Formal Analysis, Investigation, Methodology, Validation, Visualization, Writing – review & editing. MC-E: Data curation, Formal Analysis, Investigation, Methodology, Validation, Visualization, Writing – review & editing. CB: Data curation, Formal Analysis, Investigation, Methodology, Validation, Visualization, Writing – review & editing. MA: Data curation, Formal Analysis, Investigation, Methodology, Validation, Visualization, Writing – original draft. GM: Data curation, Investigation, Validation, Visualization, Writing – review & editing. CS: Data curation, Investigation, Validation, Visualization, Writing – review & editing. AP: Data curation, Formal Analysis, Investigation, Validation, Visualization, Writing – review & editing. DS: Data curation, Investigation, Validation, Visualization, Writing – review & editing. ES: Data curation, Formal Analysis, Investigation, Validation, Visualization, Writing – original draft. GA: Conceptualization, Methodology, Supervision, Validation, Visualization, Writing – original draft, Writing – review & editing. CD: Conceptualization, Funding acquisition, Methodology, Project administration, Resources, Supervision, Validation, Visualization, Writing – original draft, Writing – review & editing. SC: Conceptualization, Funding acquisition, Methodology, Project administration, Resources, Supervision, Validation, Visualization, Writing – original draft, Writing – review & editing.
