# Supplementary material for: Leukemic conversion involving RAS mutations of type 1 CALR-mutated primary myelofibrosis in a patient treated for HCV cirrhosis: a case report
Source: Front Oncol. 2023 Sep 29;13:1266996. doi: 10.3389/fonc.2023.1266996 (PMC10570518; doi:10.3389/fonc.2023.1266996)
Supplement: Supplementary file 1 [file DataSheet_1.pdf]

Material and Methods

Patient and samples

Clinical, morphological studies, and immunophenotyping data were provided by the hematologists from "Coltea Hospital". A separate written informed consent was obtained from the patient at the moment of blood and bone marrow collection for the genetic analyses performed at "Stefan S. Nicolau" Institute of Virology. The research was conducted in compliance with the Declaration of Helsinki and approved by the local ethics committee (No. 136/06.02.2017 rev. no131/18.01.2019).

Separation of cell fractions and DNA extraction

Peripheral blood mononuclear cells (PBMC) and bone marrow mononuclear cells (BMMC) were obtained by density gradient centrifugation using Ficoll-Paque PREMIUM sterile solution (GE Healthcare Life Sciences) under standard procedures. Granulocytes were separated after the collection of PBMC and red cell lysis of the pellet with hypotonic solution. CD34+ and CD3+ cells were isolated immunomagnetically from BMMC with CD34 MicroBead Kit, human, and CD3 MicroBead Kit, human, respectively (Miltenyi Biotech), according to the manufacturer's protocol. DNA was extracted from all cell fractions using PureLink™ Genomic DNA Mini Kit (ThermoFisher Scientific).

Detection of CALR exon 9 mutations

Detection of *CALR* exon 9 mutations in granulocytes, CD34+ and CD3+ cells was performed by GeneScan DNA fragment size analysis as previously described (1).

Targeted NGS

Targeted DNA sequencing of PMF patient's samples was performed with TruSight Myeloid Sequencing Panel (Illumina), an amplicon-based panel that targets the full exonic regions of 15 genes and exonic hot spots of 39 genes with known relevance for myeloid malignancies. DNA libraries were generated from 50 ng of genomic DNA per sample, following the manufacturer's guidelines. During adapter ligation, sample-specific indices were included in each library. Targeted NGS was run on the MiSeq Illumina platform using the MiSeq Reagent Kit v3.

Sequencing data were analyzed in BaseSpace™ Sequence Hub by the instrument software. FASTQ files were generated and the reads were aligned to the human hg19 reference genome. VariantStudio V2.2 (Illumina) software was used for variant annotation.

Single nucleotide polymorphism (SNP) microarray analysis

SNP microarray analysis for chromosomal aberration was performed using the the CytoScan 750K Array on GeneChip® System 3000 instrumentation platform (Affymetrix, Santa Clara, California). The CytoScan 750K Array contains 750,000 probes represented by 550,000 unique non-polymorphic probes for detection of copy number variations (CNVs) and a 200,000 SNPs for identifying copy-neutral loss of heterozygosity (CN-LOH) at the level of whole genome. This array ensures a high-resolution coverage of the exons of 526 genes involved in cancer. A total amount of 250 ng of genomic DNA was digested with NspI restriction enzyme, ligated to adaptors, and amplified with primers that bound the adaptors. PCR products were purified using magnetic beads, fragmented, labeled with biotin, and hybridized to CytoScan chip. Chromosome Analysis Suite (ChAS) v4.2.1 was employed for data analysis. Genomic data were aligned to NCBI Build 37 hg19 reference genome and reanalyzed according to NCBI (National Center for Biotechnology Information), UCSC (University of California, Santa Cruz) Genome Browser, DGV (Database of Genomic Variants), DECIPHER, and OMIM (Online Mendelian Inheritance in Man).

Reference

1.Klampfl T, Gisslinger H, Harutyunyan AS, Nivarthi H, Rumi E, Milosevic JD, et a. Somatic mutations of calreticulin in myeloproliferative neoplasms. N Engl J Med (2013) 369(25):2379-90. doi: 10.1056/NEJMoa1311347
